# Welder’s Anthrax Treated with Obiltoxaximab — Louisiana, 2024

**DOI:** 10.15585/mmwr.mm7442a1

**Published:** 2026-01-01

**Authors:** Julie M. Thompson, Eric W. Lundstrom, Lindsay D. Hein, Cari A. Beesley, Chung K. Marston, Rebecca Zaayenga, Christopher A. Gulvik, Taylor K. Paisie, Julie Hand, Chad H. Dowell, Karl Feldmann, Dawn Blackburn, Caroline A. Schrodt, William A. Bower, Katherine M. DeBord, Brian T. Richardson, Danielle Haydel, Latira Haynes, Patricia A. Yu, Yon Yu, Abigail Cocco, Maren Bell-Do, Michael Bacon, James Antonini, Nancy Burton, Zachary P. Weiner, Alex R. Hoffmaster, Marie A. de Perio, Caitlin M. Cossaboom, Theresa Sokol

**Affiliations:** ^1^Division of High-Consequence Pathogens and Pathology, National Centers for Emerging and Zoonotic Infectious Diseases, CDC; ^2^Epidemic Intelligence Service, CDC; ^3^Division of Field Studies and Engineering, National Institute for Occupational Safety and Health, CDC; ^4^Laboratory Leadership Service, CDC; ^5^Louisiana Department of Health, New Orleans, Louisiana; ^6^Office of the Director, National Institute for Occupational Safety and Health, CDC; ^7^Louisiana State Public Health Laboratory; ^8^Office of Readiness and Response, CDC; ^9^Ochsner Health, New Orleans, Louisiana; ^10^Health Effects Laboratory Division, National Institute for Occupational Safety and Health, CDC.

**Summary**

**What is already known about this topic?**

Welder’s anthrax is a serious respiratory illness caused by infection with anthrax toxin–producing *Bacillus cereus* group bacteria. Eight previous cases (six fatal) have been reported among welders or metalworkers from Louisiana and Texas.

**What is added by this report?**

In September 2024, welder’s anthrax was identified in a young, previously healthy welding apprentice in Louisiana. The patient was treated with the antitoxin obiltoxaximab, antimicrobials, and drainage of a pleural effusion, and he survived.

**What are the implications for public health practice?**

Welder’s anthrax should be considered in the differential diagnosis of pneumonia among welders or metalworkers in the southern United States. Obiltoxaximab may be used as an adjunct to antimicrobial therapy for patients with suspected welder’s anthrax. Safe work practices could help protect workers from exposure to harmful metal fumes that might predispose welders to welder’s anthrax.

## Abstract

In September 2024, the ninth documented case of welder’s anthrax was identified in a previously healthy male welder, aged 18 years, from Louisiana, who was hospitalized with pneumonia and respiratory failure requiring intubation and mechanical ventilation. Welder’s anthrax is a recently described life-threatening pneumonia caused by infection with anthrax toxin–producing *Bacillus cereus* group bacteria; risk factors for infection are not well-understood. Eight previous cases (six fatal) were reported among welders or metalworkers from Louisiana and Texas. A coordinated state and federal response facilitated use of the anthrax antitoxin obiltoxaximab (Anthim), which was administered in combination with recommended multidrug antimicrobial therapy for inhalation anthrax, including bactericidal agents and protein synthesis inhibitors. The patient’s clinical condition improved rapidly after administration of obiltoxaximab and antimicrobials and drainage of a pleural effusion. He was discharged with a tailored antibiotic regimen after a 26-day hospitalization; all of his pulmonary symptoms had resolved by his 3-month follow-up visit. An environmental investigation identified anthrax toxin genes in 28 (11.4%) of 245 soil and nonporous surface samples collected from the patient’s worksite; however, this investigation did not clearly identify host or occupational factors that contributed to his illness. Enhanced workplace safety protocols and improved engineering and administrative controls could minimize exposure to dust and welding fumes and potentially decrease environmental exposure to infectious disease agents among metalworkers. Welder’s anthrax should be considered in the differential diagnosis of pneumonia among welders and metalworkers, particularly those who live in or have worked in the southern United States. Health care providers should consult with CDC as soon as welder’s anthrax is suspected to facilitate release of anthrax countermeasures, including antitoxins such as obiltoxaximab, as adjunctive therapy.

## Introduction

Anthrax is a serious illness caused by infection with *Bacillus anthracis*, a member of the *Bacillus cereus* group. The *B. cereus* group comprises closely related, gram-positive, nonmotile, spore-forming bacteria, including, in addition to *B. anthracis*, *B. cereus* species and other newly recognized species such as *B. tropicus.*^†^ Virulence factors of *B. anthracis* include anthrax toxin and a protective capsule, encoded on plasmids pXO1 and pXO2, respectively (*1*). Persons who have contact with infected animals or animal products can become infected with *B. anthracis* (*2*). Infection with *B. anthracis* can manifest as cutaneous, gastrointestinal, or inhalation anthrax depending on how the spores enter the body (e.g., direct inoculation through skin, consumption of infected meat, or inhalation of aerosolized spores) (*2*). Inhalation anthrax is especially lethal, with estimated case fatality ranging from approximately 55% among patients who receive prompt, aggressive treatment to close to 100% among untreated cases (*2*). Current CDC guidelines for the prevention and treatment of anthrax recommend that empiric treatment regimens for nonpregnant adults with systemic anthrax include multidrug antimicrobial therapy including bactericidal agents plus a protein synthesis inhibitor. Antitoxin can be included as adjunctive therapy (*2*).

Anthraxlike illness has been observed after infection with *Bacillus* species other than *B. anthracis*. *B. cereus* is generally associated with emetic and diarrheal food poisoning. Some *B. cereus* group species, including strains of *B. tropicus*, can encode a homolog of the *B. anthracis* pXO1 plasmid containing an anthrax toxin gene (*1*,*3*,*4*). Since 1994, seven cases of severe pneumonia have been identified among immunocompetent metalworkers infected with these strains, a majority of whom were welders (*3*,*4*); an eighth case from 1996 was retrospectively identified as *B. tropicus* in a welder through whole-genome sequencing of archival isolates submitted to CDC (*1*). All previous cases occurred among welders or metalworkers from Louisiana or Texas, and the illness has been subsequently referred to as welder’s anthrax (*3*,*4*); six of the eight reported cases were fatal. Limited data indicate that these strains could be present in soil, sediments, dust, or vegetation in the southern United States (*5*). This report describes the investigation of the ninth reported case of welder’s anthrax, which occurred in a previously healthy young welder in Louisiana. This activity was reviewed by CDC, deemed not research, and was conducted consistent with applicable federal law and CDC policy.^§^

## Investigation and Findings

### Identification of Patient

On September 7, 2024, CDC and the Louisiana Department of Health (LDH) were contacted by a clinician regarding an otherwise healthy man aged 18 years from Louisiana, who was being admitted to an intensive care unit with severe pneumonia and respiratory failure that required endotracheal intubation and mechanical ventilation. The patient had developed a cough 1 week earlier. He had worked part-time as a welder for 6 months immediately preceding his illness and had no reported history of smoking, vaping, or excessive alcohol consumption. Blood cultures were positive for *B. cereus* group bacteria and, because of the patient’s pneumonia, respiratory failure, occupation, and geographic proximity to previously reported cases, welder’s anthrax was suspected (*1*,*3*,*4*) (Table).

**TABLE:** Characteristics of occupational welders and metalworkers with severe pneumonia caused by anthrax toxin–producing *Bacillus cereus* group bacteria* — Louisiana and Texas, 1994–2024

Patient	Year of diagnosis	Species of isolate	MLST	Strain	State	Occupation (duration)	Other work information	Sex	Age, yrs	Comorbidities	Outcome	Anthrax antitoxin received
1	1994^†^	*B. tropicus*	ST-78	G9241	Louisiana	Welder (unknown)	Not mentioned	M	42	None	Recovered	None
2	1996^§^	*B. tropicus*	ST-78	G9898	Louisiana	Welder (unknown)	Unknown	M	40s	Unknown	Died	None
3	2003^¶^	*B. cereus* group	ST-11	03BB102	Texas	Welder (19 yrs)	Not mentioned	M	39	Mild asthma, hypertension, and hyperlipidemia	Died	None
4	2003^¶^	*B. tropicus*	ST-78	03BB87	Texas	Metalworker (unknown)	Foundry work and grinding metal for polishing and operating machinery	M	56	Smoking	Died	None
5	2007**	*B. tropicus*	ST-78	LA2007	Louisiana	Welder (unknown)	Shipyard-related	F	47	None	Died	None
6	2011^††^	*B. cereus* group	ST-108	Elc-2	Texas	Welder (unknown)	Not mentioned	M	39	None	Died	None
7	2020^§§^	*B. cereus* group	ST-108	TX2020	Texas	Welder (10 yrs)	GMAW in fabrication shop on low-carbon mild steel	M	34	Childhood epilepsy, tobacco use, and excessive alcohol use	Died	None
8	2020^§§^	*B. tropicus*	ST-78	La2020	Louisiana	Welder (unknown)	SMAW on oil tank on new A36 mild carbon steel	M	39	Smoking, hypertension, and excessive alcohol use	Recovered	Raxibacumab
9	2024	*B. tropicus*	ST-78	LA2024	Louisiana	Welder (6 mos)	SMAW in shipyard using carbon steel and low hydrogen carbon steel electrodes	M	18	None	Recovered	Obiltoxaximab

**Abbreviations**: F = female; GMAW = gas metal arc welding; M = male; MLST = multilocus sequence typing; SMAW = shielded metal arc welding; ST = sequence type.

* The taxonomic *Bacillus cereus* group consists of closely related, gram-positive bacteria, including *B. anthracis, B. cereus, B. tropicus, B. thuringiensis*, and others. The virulence of *B. anthracis* is attributable to the pXO1 plasmid, which encodes *B. anthracis* toxin genes (edema factor, lethal factor, and protective antigen), and the pXO2 plasmid, which encodes the protective capsule. Some *B. cereus* group bacteria other than *B. anthracis* have been found to encode the pXO1 plasmid, resulting in anthraxlike illness.

^†^ https://doi.org/10.1073/pnas.0402414101

^§^ https://doi.org/10.3390/pathogens13100884

^¶^ https://doi.org/10.1086/510429

** https://doi.org/10.1128/genomea.00181-17

^††^ http://doi.org/10.5858/2011-0362-SAIR.1R

^§§^ https://doi.org/10.1093/cid/ciac535

### Treatment

The patient initially received empiric antibiotic treatment with vancomycin, meropenem, ciprofloxacin, and doxycycline. Obiltoxaximab, a monoclonal antibody anthrax antitoxin directed against the protective antigen of *B. anthracis* and indicated for treatment of inhalation anthrax, was available from the U.S. Strategic National Stockpile. CDC requested the antitoxin, which was administered to the patient 34 hours after welder’s anthrax was suspected (approximately 1 week after symptom onset). The patient’s condition improved rapidly, and intubation and mechanical ventilation was discontinued 72 hours later. He received continued intravenous antimicrobial therapy (*2*) and drainage of a pleural effusion. He was discharged with a tailored antibiotic regimen after a 26-day hospitalization. All pulmonary symptoms had resolved by his 3-month follow-up visit.

### Clinical Laboratory Analysis

Laboratory Response Network (LRN) polymerase chain reaction (PCR) testing of an isolate obtained from the patient’s blood performed by the Louisiana State Public Health Laboratory confirmed the presence of an anthrax toxin gene. CDC identified the isolate as *Bacillus tropicus* by LRN algorithm and whole genome sequencing (WGS). Gene comparisons using multilocus sequencing typing indicated sequence type 78 (ST-78) and *B. anthracis*–associated toxin genes.

### Epidemiologic Investigation

After consultation with CDC and CDC’s National Institute for Occupational Safety and Health (NIOSH), LDH interviewed the patient and representatives from his worksite^¶^ to better understand his exposure history and welding practices. For 6 months preceding his illness onset, the patient worked 4 hours per day, 4 days per week as a welding apprentice in the shipbuilding and repair industry. He conducted shielded metal arc welding** using carbon steel and low-hydrogen carbon steel electrodes both in open areas and confined spaces^††^ with limited ventilation and minimal use of personal protective equipment (PPE). No fans or ventilation systems were present in the patient’s work area, including within enclosed spaces. Interviews with worksite representatives revealed that employees work on-site for several hours each day, do not consistently wear respirators when welding, and often take breaks and eat lunch in their work area rather than using the designated break area.

### Environmental Investigation and Laboratory Analysis

After the patient recovered, LDH requested CDC’s assistance in investigating the environmental source of infection using previously described methods (*6*). A total of 245 samples were collected from the patient’s worksite, including 95 bulk soil samples from work areas, as well as 98 sterile sponge-stick^§§^ and 52 macrofoam-swab surface samples from tools or items that he possibly used (*6*). Samples were also collected from outdoor areas, tools, clothing, buildings, and break areas near the patient’s work area. All samples were stored and transported to CDC, where DNA was extracted and tested for anthrax toxin genes by LRN algorithm (*6*). A cycle threshold value <40 was considered positive. A second extraction was performed on positive samples. DNA was purified using Agencourt AMPure XP beads according to manufacturer instructions^¶¶^ for all samples with at least one negative result, and quantitative real-time PCR (qPCR) was repeated. An additional extraction was performed on positive samples and qPCR was repeated. Samples were considered qPCR-positive if two extractions were positive and inconclusive if only one extraction was positive. Culture was performed on all qPCR-positive and inconclusive samples. WGS and phylogenetic analyses were performed on culture isolates (*6*).

Among the 245 collected samples, 28 (11.4%) were positive by qPCR for anthrax toxin genes (cycle threshold range = 29.0–39.9), consisting of one swab sample from work gloves found on a vessel in an area where the patient worked (not conclusively identified as belonging to the patient), two sponge samples from a handrail and table where the patient worked, and 25 soil samples collected across the worksite, including areas where the patient worked. Five samples (2.0%, all soil) were inconclusive, and the remaining 212 (86.5%) were negative.

One isolate from a positive soil sample was successfully cultured. The genotype was ST-78; all other known ST-78 strains are *Bacillus tropicus*. WGS indicated 99.998% +/– 0.096% average nucleotide identity among genome sequences from the clinical and environmental isolates using Basic Local Alignment Search Tool (BLAST+),*** an application of the National Center for Biotechnology Information sequence similarity search program. The phylogeny further suggests their high relatedness by the short (horizontal) phylogenetic distances (Figure) and demonstrates clustering with genome sequences derived from patients from Louisiana (GCF_000832805.1, GCA_043275215.1, GCF_002007005.1, and SRR19658610), a zoo animal from Louisiana (GCF_046718865.1), the Louisiana environment (SRR19658609), a patient from Florida (GCF_000688755.1), a patient from Texas (GCF_000789315.1), and a *B. cereus* group genome with unknown origins (GCF_016027575.1).

**FIGURE:**
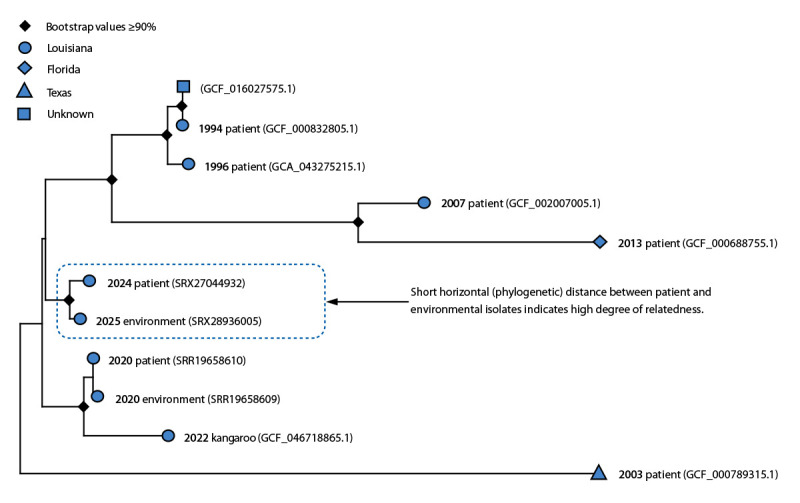
Subpanel maximum likelihood phylogenetic tree* indicating genetic distances^†^ among *Bacillus tropicus* isolates^§^^,^^¶ ^ from patients with welder’s anthrax, a zoo animal, and the environment — United States, 1994–2025 **Abbreviations**: Mbp = mega base pair; SNP = single nucleotide polymorphism. * Includes year of isolation and genome accession number corresponding to each isolate. ^†^ Reference strain = DE0099 (5.7 Mbp, Reference Sequence accession number GCF_007682035.1). ^§^ Isolates included in this phylogenetic tree were genotype sequence type 78 by multilocus sequence typing. ^¶^ SNP substitutions per core SNP from a 74% core alignment against *B. tropicus *reference strain.

## Public Health Response

CDC and LDH collaborated to provide resources and recommendations for worksites to better understand welder’s anthrax, keep employees healthy in the workplace, and follow the hierarchy of controls^†††^ (*3*). CDC suggested creating a workplace labor-management health and safety committee to discuss worksite safety concerns and implementation of a Hazard Assessment Program to recognize and address occupational hazards. To reduce exposure to soil that is possibly contaminated with *B. cereus* group organisms that produce anthrax toxins, welding surfaces and other workspaces could be kept clean and free of dust and dirt. Recommended engineering controls included wet sweeping and high efficiency particulate air filter vacuuming methods (which minimize dust creation) when cleaning workspaces and ensuring that ventilation systems are used in enclosed areas. Workers could also be educated about welder’s anthrax, trained on how to recognize the signs and symptoms, and encouraged to eat, drink, and store food only in designated break areas. The worksite could identify and provide PPE for workers based on hazard assessment findings and provide medical clearance, fit testing, and training for employees who use NIOSH-approved respirators. The degree to which these recommendations have been implemented is not known.

## Discussion

Rapid recognition and treatment of welder’s anthrax by the clinical team and an immediate and coordinated public health response likely contributed to the rapid recovery and survival of this patient. Only three of nine persons with known cases of welder’s anthrax have survived, two of whom, including this patient, received anthrax antitoxin; none of the six persons who died received antitoxin. One survivor received raxibacumab, and this case represents the first clinical use of obiltoxaximab for an anthraxlike illness.

*B. cereus* group bacteria are widely distributed in the environment, but cases of welder’s anthrax have only been documented in Texas and Louisiana, and strains containing a homolog of the pXO1 plasmid have only been isolated from the environment in Louisiana during a previous investigation (*6*). In the southern United States, cases of naturally acquired anthraxlike disease caused by these strains, including cutaneous (*7*) and gastrointestinal (*8*) forms, have been documented in humans and animals. How widely distributed these anthrax toxin–producing *B. cereus* group strains are in the United States is unclear, but ecological niche modeling (a process for estimating a spatial area conducive to the survival of these organisms) suggests that environmental conditions highly conducive to their survival are present in many states, including Arkansas, Louisiana, Mississippi, Oklahoma, and Texas (*1*).

Welding fumes are known to be harmful and to predispose welders to lung infection, in part from diminution of the respiratory immune response to infections caused by metal fumes (*3*,*9*). The reason that this previously healthy young man was the only worker to become ill, despite the detection of anthrax toxin genes in multiple environmental samples from his worksite, is unclear. Before this case, one half of reported welder’s anthrax cases occurred among persons with comorbidities and health risk factors, including smoking or tobacco use, excessive alcohol use, hypertension, and asthma. This patient had none of these comorbidities or health risk factors and had a relatively brief history of working as a welder. However, shielded metal arc welding produces more fumes than other types of welding do, which might facilitate respiratory infection via immune system suppression (*9*). Positioning, skill level, use of exhaust ventilation systems, and time spent welding could also contribute to the duration and intensity of welding fume exposures among welders (*10*). Improved engineering and administrative controls and use of PPE can reduce metal fume and dust exposure, potentially reducing risk for welder’s anthrax and other respiratory infections.

### Limitations

The findings in this report are subject to at least two limitations. First, the patient was unable to provide the clothing, gloves, and boots worn during work, and whether the specific tools and welding equipment present on the worksite during the environmental investigation were used by the patient is unknown. This investigation detected the presence of anthrax toxin genes around this worksite; however, testing items used by the patient for the presence of anthrax toxin genes was not possible because clothing and footwear were not retained by the patient. Due to lack of workplace exposure data, assessment of the patient’s occupational exposure history was limited to interviews and observation of his workplace after his illness, limiting the ability to characterize specific occupational hazards or work practices that might have contributed to the development of welder’s anthrax.

### Implications for Public Health Practice

Infection with anthrax toxin–producing *B. cereus* group bacteria should be considered in the differential diagnosis when evaluating welders or metalworkers with pneumonia, particularly in the southern United States. Vaccination against anthrax has not been studied among welders, and the role of vaccination as a medical countermeasure for this population is not currently recognized or understood (*2*,*3*). When managing a patient with suspected welder’s anthrax, clinicians may consult current guidelines for the treatment of inhalation anthrax (*2*), which include the use of multidrug therapy with bactericidal agents and protein synthesis inhibitors (e.g., ciprofloxacin and clindamycin), bearing in mind that *B. cereus* group bacilli are often resistant to penicillins and cephalosporins. Clinicians should also inform jurisdictional public health departments of suspected cases and consult with CDC as soon as welder’s anthrax is suspected to facilitate the release of anthrax-associated medical countermeasures (e.g., antitoxins such as obiltoxaximab) from the U.S. Strategic National Stockpile. To help prevent infections, employers of welders and metalworkers can follow best practices to minimize workplace exposure to welding fumes and gases, as well as soil and dust exposure in worksite areas where the bacteria might be present. Ongoing study and analysis of environmental, occupational, and host factors associated with welder’s anthrax are needed to identify causes that can guide development and implementation of definitive prevention and control measures.

## Footnotes

* These authors contributed equally to this report.

† *Bacillus tropicus* (formerly *B. cereus*) is a newly recognized *Bacillus cereus* group species. Proposal of nine novel species of the Bacillus cereus group | Microbiology Society

§ 45 C.F.R. part 46.102(l)(2), 21 C.F.R. part 56; 42 U.S.C. Sect. 241(d); 5 U.S.C. Sect. 552a; 44 U.S.C. Sect. 3501 et seq.

¶ Questionnaires used during patient and worksite interviews included information on demographic characteristics, medical history (including history of vaccination against anthrax), tobacco product use, alcohol use, travel history, military service, occupational history, history of welding activities (including welding processes and metals used), workplace exposures (e.g., solvents, smoke, dust, and asbestos), personal protective equipment use (including respiratory protection), food consumption in the workplace, and cleaning and decontamination activities (e.g., handwashing, changing clothes and shoes, dry sweeping, and dust exposure).

** Shielded metal arc welding, also called stick welding, joins steel plates using an electric current and can expose workers to fumes containing various metals (e.g., manganese, chromium, and nickel). This type of welding and flux core arc welding tend to produce more welding fumes than do gas metal arc welding or tungsten inert gas welding. Occupational Safety and Health Administration Fact Sheet: Controlling Hazardous Fumes and Gases During Welding

†† Confined spaces are enclosed or partially enclosed areas large enough for a worker to occupy despite having few entry or exit points and limited ventilation. Confined Spaces | Occupational Safety and Health Administration

§§ Macrofoam swabs and cellulose sponges were used to sample nonporous surfaces according to established protocols for *B. anthracis* sampling procedures. Surface Sampling Procedures for *Bacillus anthracis* Spores from Smooth, Non-porous Surfaces | Anthrax | CDC

¶¶ DNA was purified using Agencourt AMPure XP beads according to manufacturer instructions. Agencourt AMPure XP PCR Purification

*** BLAST+ is a sequence-similarity search program available online through the National Institutes of Health National Center for Biotechnology Information website, and BLAST+ is an application of this National Center for Biotechnology Information search program. BLAST+: architecture and applications | BMC Bioinformatics

††† The hierarchy of controls includes five levels of actions to reduce or remove hazards in the workplace. The preferred order of action based on general effectiveness is 1) elimination (removal of the hazard), 2) substitution (use of a safer alternative), 3) engineering controls (reducing contact of workers with hazard), 4) administrative controls (reducing duration, frequency, or intensity of exposures), and 5) PPE (use of equipment to minimize exposure to hazards). Using this hierarchy of controls can lower worker exposures and reduce risk for illness or injury. About Hierarchy of Controls | Hierarchy of Controls | CDC
